# Ritualization as Alternative Approach to the Spiritual Dimension of Palliative Care: A Concept Analysis

**DOI:** 10.1007/s10943-019-00792-z

**Published:** 2019-04-06

**Authors:** Kim van der Weegen, Martin Hoondert, Madeleine Timmermann, Agnes van der Heide

**Affiliations:** 1 Department of Public Health, Erasmus MC, P.O. Box: 2040, 3000 CA Rotterdam, The Netherlands; 2 Department of Culture Studies, Tilburg University, Tilburg, The Netherlands; 3 Stichting Groenhuysen, Roosendaal, The Netherlands

**Keywords:** Ritual, Ritualization, Palliative care, End of life, Spiritual care

## Abstract

The spiritual dimension is considered to be a central component of palliative care. However, healthcare professionals have difficulties incorporating the spiritual dimension into their everyday practice. We propose a new approach by looking beyond the mere functionality of care practices. Rituals and ritualized practices can serve to express and communicate meanings and values. This article explores how ritualized practices have the ability to open up space for the spiritual dimension of care in the context of palliative care.

## Introduction

In both theory and practice, the spiritual dimension of palliative care is receiving increasing attention. Studies in different countries worldwide show that both patients and healthcare professionals view spiritual care as an essential component of treatment and care practice (Giezendanner et al. 2017; Wittenberg et al. 2016). Despite the importance that is attributed to spiritual care, putting it into practice proves to be difficult. Different models, guidelines, and teaching programs are available nowadays to help healthcare professionals in providing spiritual care. The effects of these tools are not always clear or measurable. This study is approaching the spiritual dimension from a different angle, through the lens of rituals. We aim to clarify the connection between the spiritual dimension of palliative care and rituals and show how this approach is relevant for the spiritual dimension of care. First, challenges surrounding the concept of spirituality will be clarified, which is needed to understand current trends in studies focussing on spiritual care. Next, the concepts of rituals and ritualization will be introduced. Finally, ritual will be elaborated on as a way of approaching the spiritual dimension of palliative care.

## Defining Spirituality

Spirituality is a term that is used in different contexts of care, but it is not always clear what spirituality encompasses. Although the World Health Organization (2018) acknowledges the importance of the spiritual dimension of care in their definition of palliative care, they do not define the concept. Because of the many associations connected to the concept of spirituality, it is important to clarify what is meant by spirituality for the purpose of this article. Religion can be regarded as spirituality, although religion often implies an organization in a system or tradition with certain values, practices, and beliefs (Edwards et al. 2010: 1; Grimes 2014: 1997; Tanyi 2002: 502; Vachon et al. 2009: 53). Religion is characterized by boundaries, as opposed to spirituality, which is a more wide-ranging concept of which the boundaries are difficult to determine (Edwards et al. 2010: 1; Tanyi 2002: 502; Vachon et al. 2009: 53). Because spirituality does not necessarily involve religion and can mean different things to different people, a broad definition will be used in this article:

Spirituality is the dynamic dimension of human life that relates to the way persons (individual and community) experience, express and/or seek meaning, purpose and transcendence, and the way they connect to the moment, to self, to others, to nature, to the significant and/or the sacred (Nolan et al. 2011: 88).

This definition was developed in the context of palliative care in a European consensus report. The aim was to come to agreement about what spirituality in palliative care involves. The definition closely resembles the definition that was developed in an influential American consensus report by Puchalski et al. (2009).

In a simplified way, the concept of spirituality is often characterized as a search for meaning. While there is truth in this characterization, Kellehear (2000) argues that in the context of health care, and more specific palliative care, this description competes with other domains of care, such as psychological care. A similar line of thought was developed by Bradshaw (1996) in her critique on the secularization of end-of-life care. She claims that by regarding spirituality as a personal and psychological search for meaning there is a risk of not addressing the true needs of patients on the spiritual level. Therefore, it is important to pay attention to the connection that the concepts of transcendence and the sacred have to meaning making. Both concepts are present in the definition of spirituality and are essential to distinguish the spiritual dimension of care from the psychological and social dimensions of care.

Kellehear builds his model of spiritual needs on the desire of human beings to transcend hardship and suffering. He states that people have a need: ‘[…] to seek and find a meaning beyond their current suffering that allows them to make sense of that situation’ (2000: 150). Transcendence refers to something that is beyond immediate experiences or situations. That which brings about an experience is transcendent to that experience. So if, for example, a nurse feels good about helping a patient in need, the patient is not the means to an end, to make the nurse feel good about herself. The patient is transcendent to the experience of the nurse. If the patient were the means to an end, to make the nurse experience a good feeling, the experience would lose its significance for the nurse. Transcendence is closely related to the concept of sacrality. It is important to emphasize that by using the term sacrality we do not exclusively refer to the religious sacred, but to what Evans (2003) calls the set apart sacred, which can also be found in experiencing nature, sport, or art, amongst others. We follow Lynch (2012a, b) in his cultural sociology of the sacred, referring to the moral or guiding values on which people build their lives. Think of values such as empathy and care. The sacred is set apart because it transcends the physical reality of everyday life. The moral or guiding values that we hold sacred seem natural and fixed, so we are often not aware of them. However  they are products of culture and history that are constantly being negotiated and recreated. Sacred values do not just direct human actions, but also our emotions towards objects, symbols, and actions.

Spirituality is about what gives meaning and value to life. Identifying meaning and value in life is difficult, because it is based on sacred values that transcend our everyday reality. Here the problem of defining spirituality comes in. In the end, there is no definition of spirituality that can fully comprise its meaning. Not being able to fully grasp the concept of spirituality in a concise and concrete description is one of the difficulties in incorporating the spiritual dimension of care into the everyday practice of palliative care.

## The Spiritual Dimension of Palliative Care

The concept of spirituality is an important contemporary topic in health care. In both practice and theory, the terms spiritual care and spiritual dimension of care are used interchangeably. A large amount of research and development is going on around the topic of spirituality in health care. It is beyond the scope of this article to give an overview of all research and developments. We will only briefly discuss some developments from the field of palliative care to illustrate why a different approach to the spiritual dimension of care might be useful.

Patients who are diagnosed with an incurable illness are facing difficult emotions and thoughts. In the face of death, spiritual concerns rise to the surface. Patients express a need for support in alleviating spiritual pain and in addressing issues of meaning (Sinclair et al. 2006). In a literature review by Edwards et al. (2010: 13), it was found that specific elements that are important to patients are: finishing business, forgiveness and reconciliation, letting go and acceptance, life review and reminiscence, involvement and control, and a positive outlook. Besides these elements, the review also found that next to the need to talk about death and dying, patients longed for ordinariness and normality, and talking about ‘regular’ things.

According to healthcare professionals, spirituality should be addressed by connecting with a patient, being there, listening, and showing empathy. An important aspect is that nothing needs to be done (Sinclair et al. 2006; Vermandere et al. 2013). This approach to spirituality in palliative care and health care in general resembles how care is approached in the field of ethics of care. Both Baart and Vosman (2011) in the Theory of Presence and Van Heijst (2011) in what she calls Professional Loving Care emphasize attentiveness and the care relationship as core features of care. When it comes to spirituality, Edwards et al. (2010: 12–3) also place the relational character of care in a central position. Meeting the spiritual needs of patients should not be addressed in a task-oriented fashion but in the context of a relationship. Bradshaw (1996) expresses concerns about current developments in spiritual care. She states that nowadays there is an emphasis on acting in the form of counselling, talking, and self-expression. This turns care into a technique instead of a genuine compassionate relationship, which is undesirable.

Despite the recognition of the importance of addressing spirituality in health care, professionals find it difficult to incorporate it in their daily practice. Healthcare professionals experience a lack of knowledge, lack of skills, a high workload, and lack of time as important barriers for addressing spirituality (Edwards et al. 2010; Giezendanner et al. 2017; Wittenberg et al. 2016). The lack of clarity about the concept of spirituality is also an impeding factor (Wittenberg et al. 2016). In an effort to assist healthcare professionals in addressing spirituality, guidelines, screening and assessment tools, training programs, and other interventions are being developed and implemented. But by confining spirituality to a technique, tool, or guideline, the impalpable character and core of spirituality is at risk of getting lost.

The same trend can be seen in literature in which spiritual care is presented as a distinct type of care, with its own diagnoses and interventions. Like other aspects of patient care, there is a push towards scientifically informed or evidence-based spiritual care. Although there are empirical scientific studies on spiritual care, many of them are criticized on the basis of their methodology and issues with the operationalization of their definitions (Balboni et al. 2017; Edwards et al. 2010; Steinhauser et al. 2017). Current developments in research on spirituality and professional health care show that the two are difficult to reconcile. At this point, the paradoxical character of evidence-based spiritual care comes into play (O’Connor 2002). The spiritual is deeply personal and can never be fully grasped. Professional health care, on the other hand, is provided systematically within the field of medicine. Concrete actions such as systematically observing, analysing, measuring, diagnosing, and treating are difficult to reconcile with spirituality. The risk of using tools and screening instruments is that they train the healthcare professional to focus on spiritual problems rather than spirituality as a core aspect of humanity. Showing that care can still be valuable when no problems can be solved, Van Heijst (2011) calls on the concept of expressionate acts, originally introduced by Rudolf Ginters as *ausdruckshandlung*. Through expressionate acts, connectedness and the underlining that people are precious by principle are values that can be realized. This is exactly what becomes clear in end-of-life care.

Spiritual care should not be about identifying and treating problems, but about being there and connecting to the patient. Spirituality cannot be separated from other aspects that define us as human beings, such as our bodies, our minds, the people, and the world around us. They are all interrelated. Spiritual care should therefore not be treated as a separate form of care but should form an integral part of all aspects of care. Instead of using the term ‘spiritual care’, we will speak about ‘the spiritual dimension of care’. We propose an alternative way to approach the spiritual dimension of care, through the practice of rituals. In order to clarify this alternative approach, it is important to clarify what is meant by rituals in relation to palliative care, or health care in general.

## Defining Ritual

Ritual is a broad concept, as can be said about spirituality. Scholars from disciplines such as religious studies, anthropology, liturgical studies, and theatre and performance studies have all studied rituals from their perspectives. Together they form the field of ritual studies which has developed itself over the past 40 years (Post 2015). Rituals can be found anywhere where people are and thus in healthcare settings as well. In everyday language, the term ritual can have a negative connotation when it is used to describe repetitive and useless acts. In health care, the term ritual is sometimes used to refer to an act that is deemed inefficient and ineffective. This view corresponds with the current focus on evidence-based practice in the healthcare sector. Another approach, the approach of ritual studies, focusses on rituals as a cultural phenomenon and focusses on the structures, meanings, and functions of rituals. It is the latter approach that we will follow.

Formally defining rituals can lead to an idea that there are clear-cut boundaries to what makes up a ritual. In reality, these boundaries are not always clear-cut and can differ depending on the context that is being studied (Grimes 2014: 196). However, there are common criteria, characteristics, or qualities that can be ascribed to rituals, to work towards a so-called polythetic definition of ritual. This approach to ritual is derived from Snoek (2006). A polythetic definition works with a set of characteristics which may be applied but do not necessarily have to, as opposed to a monothetic definition that uses exclusive criteria that all have to apply. A broad and polythetic definition that would suit the context of rituals and the spiritual dimension of care is a definition that Paul Post developed based on Ronald Grimes.

Ritual is a more or less repeatable sequence of action units which take on a symbolic dimension through formalization, stylization, and their situation in place and time. On the one hand, individuals and groups express their ideas and ideals, their mentalities and identities through these rituals, on the other hand the ritual actions shape, foster, and transform these ideas, mentalities and identities (Post 2015: 7).

Characteristics that can be derived from this definition are repetition, enactment, symbolism, formalization, and stylization. The second part of the definition focusses on possible functions of rituals such as the expressive, social, and ethical functions that rituals can have. Despite the use of a definition and characteristics of a ritual, it is not straightforward to identify an act as a ritual. Grimes (1990) states that when an act becomes dense with ritual characteristics one can speak of ritualization or even a ritual. Whether an act is acknowledged as a ritual is not a matter of definition, but a cultural issue. Focussing on everyday practices in the field of palliative care, only a small number of activities can be formally labelled a ritual. However, there are many care practices which have ritual characteristics or functions. Consequently the focus within this article is placed on the processes of ritualization that take place in everyday care practices in the field of palliative care. The process of ritualization can be described as consciously or unconsciously adding a ritual dimension to the practice of care.

Although not specifically mentioned in the above-cited definition of ritual, it is important to address the association between ritual and religion. Many of the formal rituals people perform can be linked to religion. According to Grimes (2014: 196–197), ritual, spirituality, and religion are interconnected terms and have to be approached as such. Because this article focusses on rituals in care practices, and not necessarily on formal rituals taking place within society as a whole, institutionalized religion plays less of a role in this context.

## Ritual as Practice

In the context of palliative care, everyday practices can be studied through the lens of rituals or processes of ritualization. Ritualization is: ‘[…] a way of acting that distinguishes itself from other ways of acting in the very way it does what it does; moreover, it makes this distinction for specific purposes’ (Bell 1997: 81). The way a ritualized act is performed distinguishes it from other acts; it gives the act a special significance. The degree of ritualization is one of the factors that determine whether an act is a ritual. When lower degrees of ritualization are at play, we can speak of ritual-like activities. Therefore we make a distinction between rituals and ritual-like activities. Ritual-like activities are seemingly normal everyday activities that are bearing a non-routine significance (Bell 1997: 166). People often do not identify an activity as ritual-like because the activities, to them, seem like the natural or appropriate thing to do in a situation. Bell (1997: 168) refers to this phenomenon as the naturalization of ritual-like activities. It is therefore important to keep in mind the characteristics and functions of ritual, such as: symbolism, formalization, and stylization when studying processes of ritualization.

Because we focus on processes of ritualization in everyday practices, a practice approach to ritual is applied. Bell (1997: 76–81) describes how practice theory sees human activities as creative strategies to reproduce and reshape social and cultural environments. According to this approach ritual is conceived as a special kind of practice, a practice that involves ritualization. Consequently Bell proposes a systematic framework for analysing ritual as practice. The framework addresses how a particular community or culture ritualizes and what characteristics of acting make strategic distinctions between these acts and others. Also the framework addresses when and why ritualization is deemed to be the appropriate way to act. Following this practice approach to ritual, it is important to study ritual in its real context, as an act that is embedded in a larger spectrum of acting (Bell 1997: 81). In the context of health care, many acts come together that cannot be studied separately when focussing on ritualization. Think of the numerous medical acts, professional care acts, informal care acts, and the personal acts and routines of patients, all taking place within the same context. This whole spectrum of acts determines what practice looks like in specific situations and thus how ritualization takes shape. Patients, family members, and healthcare professionals take on active roles in reacting to and defining practices. They determine, often unaware, when and how ritualization takes place. This way care activities can move from routine activities to ritual-like activities in certain contexts.

According to Bell, ‘[…] the study of ritual as practice has meant a basic shift from looking at activity as the expression of cultural patterns to looking at it as that which makes and harbours such patterns. In this view, ritual is more complex than the mere communication of meanings and values; it is a set of activities that construct particular types of meanings and values in specific ways’ (Bell 1997: 82). If we now go back to the definition of spirituality and its core features: meaning, sacrality, and transcendence, the connection becomes more obvious. Ritualized practices produce something we cannot fully grasp; it transcends the everyday reality of the situation. We now enter the domain of spirituality. Spirituality is about meaning that goes beyond our immediate reality; it is about the sacred values we build our lives around. Medical activities and care activities are not just functional but have the ability to produce meaning. The spiritual dimension of care is produced by its practice. Care is not just an act but it involves a (sacred) value that is produced through (inter)action. This becomes especially evident in the context of palliative care.

## Ritual and the Spiritual Dimension of Palliative Care

Rituals take place throughout our lives, but they become more visible during pivotal moments. Being confronted with illness and death is a defining moment in life. Emotions and questions rise to the surface which cannot all be dealt with in a rational way. Rituals can help to provide a sense of meaning in situations we do not fully understand. Palliative care settings provide rich ground for rituals. When nothing can be done in terms of curing illness, rituals can provide an important source of meaning.

In every culture, there are numerous formal rituals surrounding illness and death. Since palliative care deals with illness and death, some of these formal rituals also take place in this setting. Although not the primary focus of this study, an example of a formal death ritual would clarify what rituals can do in these situations. A commonly performed ritual surrounding death is a commemoration ceremony. This type of ritual can also be found in the context of palliative care. Running et al. (2008) describe a commemoration ceremony that is performed in a hospice setting as a grief ritual for hospice staff. This ceremony has a therapeutic function for the hospice staff and is seen as important to continue doing their work in a compassionate way. There are several elements that are recurring in this type of grief ritual. A first element is the use of symbolism, in the form of photos, objects belonging to the deceased, candles, music, or texts. Another element is creating a safe and structured moment to express emotions. This way the ritual can help to get in touch with ones emotions but also channel them so they do not become overwhelming. Finally the act of reminiscing is an important and recurring element. Sharing stories about the deceased can be therapeutic and emphasize a continuing bond with the deceased (Klass et al. 1996). This formal ritual shows how the spiritual dimension of care is addressed by healthcare professionals through practice. Although there is no direct interaction between patients and professionals in this situation, the act is an important part of the whole spectrum of acts within the hospice setting. The symbolic act raises awareness that the work the nurses do is meaningful. Because hospice nurses guide their patients through the proces of dying, being able to look back on a peaceful death gives a sense of satisfaction. By pausing for a moment and commemorate, the meaning and value of their work is emphasized and helps them to continue their work with other patients.

In the context of palliative care, there are numerous examples of ritualized acts that provide a sense of meaning to patients, families, and healthcare professionals. This can range from small and seemingly simple acts to bigger and more structured acts. Van der Geest (2005: 140) describes how the act of fluffing up a patient’s pillow by a nurse is not just a functional act of making the patient more comfortable. This small and simple act also shows that the nurse is concerned with the patient, which in return might fill the patient with a positive feeling. The spiritual dimension of care is present in this seemingly small act. It exemplifies a caring relationship and shows that the patient is valuable to the nurse.

An example of a bigger and more structured ritual-like act is post-mortem care that is provided by nurses. In his study on nursing rituals, Wolf (1988: 61–62) describes post-mortem care as a therapeutic nursing ritual. Post-mortem care is not simply delivered in a standardized way, but on the basis of the nurses’ shared beliefs, values, and knowledge on how to care for a deceased patient. During post-mortem care, the patient is still seen as present. Care is provided in a respectful manner, with awareness of the patient’s humanness. Gently traces of previous suffering are washed away and removed, both from the patient and from the room. Symbolically, the ritual ends the nurses’ moral responsibilities towards the patient. The ritual is therapeutic because it helps the nurses to deal with the harsh reality of death. Again, this act shows the sacredness of human body and life because the patient is cared for in the same way before and after death. The act shows the connection between the nurses and the patient and the value attributed to the process of a good death.

Although the above-mentioned acts might not be evidence-based interventions of which effects can be measured, they do hold up sacred values, as understood according to the cultural sociological approach by Lynch (2012a, b). These actions refer to the sacred value of human life, the sacred value of the dying process and human connectedness. Exactly these types of actions create space for the spiritual dimension of care, a space in which meaning and value can be produced and communicated.

## Conclusion

A current trend in research and practice is to confine the complex concept of spirituality into models, tools, and methods that focus on intervention. This approach is understandable since it fits in with existing ways of providing health care. However, the core elements of what the spiritual dimension of care should be is at risk of getting lost in the process. The spiritual dimension of care is about being there and connecting to a patient, not about intervening. Therefore, the spiritual dimension of care is connected to all aspects of care. We propose not to approach spiritual care as a separate activity but as a part of care practice as a whole. Care practices can gain special significance through a process of ritualization. As a result, rituals and ritual-like acts create space for the spiritual dimension of care. In the context of palliative care, the spiritual dimension of care becomes especially important. When illness cannot be cured, the focus should be on what is of meaning and value in the time that is left.

Processes of ritualization have not been studied earlier in relation to the spiritual dimension of care. It would be valuable to further explore how rituals and ritual-like acts can help healthcare professionals to incorporate the spiritual dimension of care into their daily practices. In this article, we have justified that a practice-oriented approach to spirituality is a way to further incorporate the spiritual dimension into palliative care. By making health care professionals aware of the symbolic dimension of their care practices, they can learn to more consciously incorporate the spiritual dimension of care in their everyday practice.
